# Resolving single molecule structures with Nitrogen-vacancy centers in diamond

**DOI:** 10.1038/srep11007

**Published:** 2015-06-05

**Authors:** Matthias Kost, Jianming Cai, Martin B. Plenio

**Affiliations:** 1 Institut für Theoretische Physik, Albert-Einstein Allee 11, Universität Ulm, 89069 Ulm, Germany; 2 Center for Integrated Quantum Science and Technology, Universität Ulm, 89069 Ulm, Germany; 3 School of Physics, Huazhong University of Science and Technology, Wuhan 430074, China

## Abstract

We present theoretical proposals for two-dimensional nuclear magnetic resonance spectroscopy protocols based on Nitrogen-vacancy (NV) centers in diamond that are strongly coupled to the target nuclei. Continuous microwave and radio-frequency driving fields together with magnetic field gradients achieve Hartmann-Hahn resonances between NV spin sensor and selected nuclei for control of nuclear spins and subsequent measurement of their polarization dynamics. The strong coupling between the NV sensor and the nuclei facilitates coherence control of nuclear spins and relaxes the requirement of nuclear spin polarization to achieve strong signals and therefore reduced measurement times. Additionally, we employ a singular value thresholding matrix completion algorithm to further reduce the amount of data required to permit the identification of key features in the spectra of strongly sub-sampled data. We illustrate the potential of this combined approach by applying the protocol to a shallowly implanted NV center addressing a small amino acid, alanine, to target specific hydrogen nuclei and to identify the corresponding peaks in their spectra.

Nuclear magnetic resonance spectroscopy (NMR) allows for the structure determination of molecules and proteins and therefore contributes fundamentally to the advancement of the biological sciences. This structural information is obtained by probing the target of investigation, typically a large molecular ensemble, by means of multiple radio frequency pulses and measuring their response. This response is then mapped to multi-dimensional spectra which encode the dynamical properties of the system and therefore of the interactions between its constituent nuclear spins^1^,^2^,^3^. These data in turn permit the reconstruction of their mutual distances and from this of the entire molecular structure. Due to the minute size of the nuclear magnetic moments compounded by the tiny polarization of these nuclear spins at room temperature, even in very strong magnetic fields, large ensembles (at least 10^12^ molecules) are required in order to extract a measurable signal. Thus NMR is susceptible to inhomogenous broadening and can only deliver ensemble information while the structure and dynamics of individual specimens remain hidden from observation^4^,^5^,^6^.

The recent progress in the control of a single electron spin in Nitrogen-vacancy (NV) centers in diamond offers a new perspective here as it becomes possible to use optically detected magnetic resonance^7^,^8^ to read out the effect of smallest magnetic fields^9^,^10^,^11^,^12^. Recent theoretical investigations^13^,^14^ have suggested that shallowly implanted NV centers^15^,^16^ in conjunction with dynamical decoupling methods^17^,^18^,^19^,^20^ should be able to detect and locate individual nuclear spins above the diamond surface. Subsequent experimental work has achieved the observation of ensembles of nuclear spins outside of diamond^21^,^22^,^23^ and more recently the detection of single digit numbers of silicon nuclear spins with a sensitivity that is sufficient to identify even individual nuclear spins^24^. Remarkably, this experiment has also demonstrated a new detection regime in which the NV center (that is a quantum sensor) couples more strongly to the external targetted nuclear spins than these spins are coupling to their neighbours. In consequence, even an unpolarised sample can lead to a signal with full contrast as the random nuclear spin flip-flops are to slow to average out the NV-nuclear spin interaction. This alone results in a million-fold improvement of sensitivity over standard NMR^24^.

Beyond this remarkable enhancement of the NMR signal, it is important to realize that this new detection regime offers further opportunities beyond the capabilities of standard NMR. In addition to the manipulation and probing of nuclear spins by means of external radio-frequency fields we are now in a position to control the properties of the NV center such as to tailor its response and, crucially, via its strong interaction with nuclei also to obtain an additional handle for the manipulation of the nuclear spins. In this work we take advantage of this potential and demonstrate the usefulness of single molecule NMR by means of strongly coupled NV centers.

The large amount of required data and the associated long measurement times represent a challenge that is common to both ensemble NMR and single molecule NMR measurements. However, the spectral information that is being obtained in any NMR experiment possesses underlying structure, determined by the Hamiltonian that describes the mutual interactions of the nuclear spins in the molecule, which makes the system sparse in a suitable basis. This fact can be exploited by non-linear reconstruction methods from signal processing that are designed to unravel such structures without prior knowledge with the minimal possible number of measurements. Indeed, measuring only a small subset of all accessible data points can be shown to allow for the reliable reconstruction of spectral information as it is required in NMR protocols.

In this work we are combining one such approach, matrix completion^25^,^26^ (see^27^ for applications of the related but distinct compressive sensing to bulk NMR), and NMR spectroscopy with an NV spin quantum sensor in the strong coupling regime to devise a new regime of single molecule NMR that may provide a novel route towards the long-term goal of elucidating the structure of individual molecules and proteins.

This article proceeds as follows: The first section begins with a numerical discussion of single molecule two-dimensional-NMR (COSY) by means of strongly coupled NV centers in diamond. It also introduces techniques of selective polarization enabled by strong coupling to enhance the contrast in the NMR spectra and presents realistic numerical simulations of the envisaged set-up. A second part of this section presents protocols that employ an NV quantum sensor that is strongly coupled to external nuclei to resolve inter-molecular couplings in 2D correlation spectroscopy at the single molecule level even without previous polarisation of the target molecule. For illustration we apply these schemes numerically to simulations of the application of our scheme to a simple bio-molecule, alanine. In a final section this article discusses and demonstrates the power of matrix completion to reduce the data taking effort and hence experiment time in single molecule NMR by means of numerical examples.

## Basic principles of 2D NV spectroscopy in the strong coupling limit

In this section, we will introduce the basic principles of 2D correlation spectroscopy (COSY) which we will then use as a test case for the theoretical study of the exploitation of the strong coupling regime of an NV quantum sensor with target nuclei.

**Figure 1:**
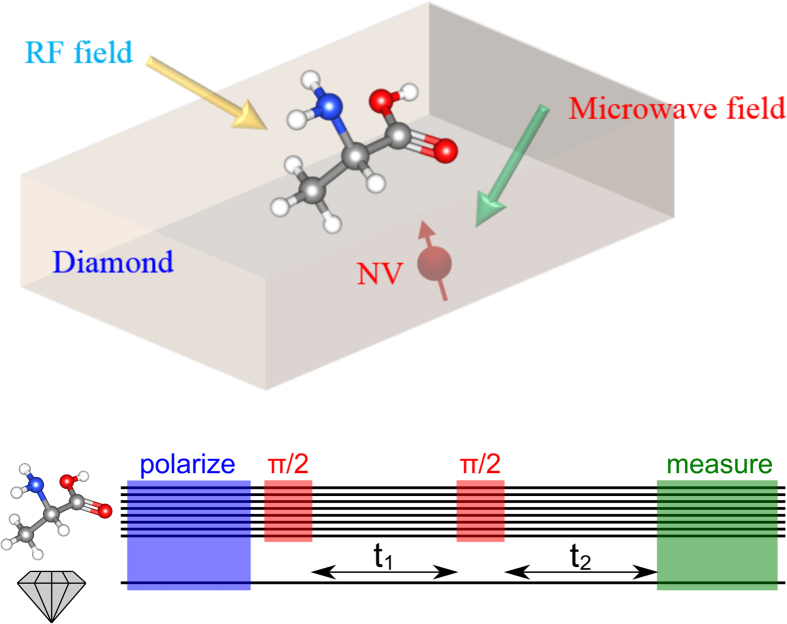
Two-dimensional correlation spectroscopy (COSY) pulse sequence. The nuclei (upper row of horizontal lines) are initially polarized by a NV spin sensor (blue box) and prepared into a coherent superposition by the subsequent application of a radio-frequency *π*/2-pulse (red box) on the nuclei only. The nuclei then undergo a free evolution for time *t*_1_ followed by a second radio-frequency *π*/2-pulse (red box). After a further free evolution time *t*_2_, the polarization of nuclear spins is measured by a NV spin sensor (green box). During the free evolution times, the interaction between the NV center electron spin and the nuclei is eliminated by transferring the NV spin to the *m*_*s*_ = 0 ground state.

*Elements of COSY in the strong coupling limit –* The basic variant of COSY employs two pulses separated by an incremented delay *t*_1_ where the response of the system is measured after a time interval *t*_2_ (see red and green sections of Fig. 1). The experimental data collected in this manner is typically Fourier transformed in both dimensions (*t*_1_ and *t*_2_) to yield the frequency domain COSY spectrum. The resulting diagonal peaks of the signal in frequency space refer to the eigenfrequencies of the nuclear spin system, and the off-diagonal peaks indicate polarization exchange between pairs of nuclei in the target molecule.

In COSY, as is common to standard NMR protocols executed on large ensembles or in the weak coupling regime, the measured signal will be proportional to the nuclear spin polarisation. In the strong coupling regime we can enhance the collected signal by making use of the possibility of achieving hyperpolarization, i.e. nuclear spin polarization far beyond thermal equilibrium conditions, of all or a selected few of the nuclei by means of dynamical nuclear spin polarization via the NV centers^28^,^29^. Hyperpolarization of specific sets of nuclei can be achieved by selection in frequency space via magnetic field gradients, together with radio-frequency pulses (summarized as the blue shaded part in Fig. 1). We remark that it is possible to avoid the use of radio-frequency pulses by leveraging the strong coupling between the NV spin sensor and the nuclei to achieve indirect control over the nuclei by means of microwave control of the NV center electron spin^30^. The measurement of nuclear spin polarization will finally be achieved by the same NV quantum sensor^31^.

Neglecting rapidly oscillating terms, that is under the rotating wave approximation, the dynamics of the NV center and the nuclei in an interaction picture with respect to *H*_0_ = * ω*_*MW*_*σ*_*z*_, where *ħ* = 1 and * ω*_*MW*_ is the frequency of the microwave drive, is governed by the effective Hamiltonian


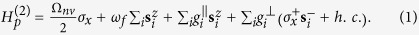


Here Ω_*nv*_ is the effective Rabi frequency of the microwave field applied to the electronic 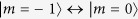 transition between of the NV spin in the electronic ground state, and *σ*_*x*_ is the Pauli operator in the corresponding subspace spanned by {|*m* = −1〉, |*m* = 0〉}, 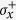 is the raising operator in the eigenbasis of *σ*_*x*_ and 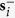 represents the nuclear spin lowering operators. Nuclear spin polarization can now be achieved by exploiting a Hartmann-Hahn condition between the Rabi frequency of the driven electron spin of the NV center and the Larmor frequency of the nuclear spins^28^. It has been observed before^31^ that the polarization procedure can be enhanced if the internuclear coupling is reduced during the polarization and the measurement process. This can be achieved by applying radio frequency fields which are most effective for a detuning Δ_*p*_ from the Larmor frequency of the nuclei and a field strength Ω_*p*_ that satisfy 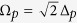. The result is a new effective nuclei energy scale 
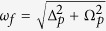
31. In this case the Hartmann-Hahn condition changes as it needs to account for the detuning and driving fields to find


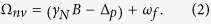


If this conditions is fulfilled, electron-nuclear spin-flip-flop processes lead to polarization exchange between the NV center spin and the nuclei and result in the efficient polarization of the nuclear spins. Alternatively to the radio-frequency field decoupling, a strong magnetic gradient may be applied in which case even identical nuclear spin species will have different Larmor frequencies. While this may require a sweep across the frequency range, as implemented e.g. in the integrated solid effect^29^,^32^, it also opens the opportunity to allow polarisation to dominate for specific nuclei.

Finally, the measurement of the polarization of the nuclei is also achieved by the NV center. Indeed, we can infer the average polarization of the nuclei from the measurement signal of the NV center spin by^31^


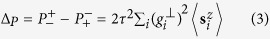


where 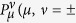) is the population of the NV center in state |*v*〉 after being initialized in state |*μ*〉 and a free evolution determined by eq. (1) for a period of time 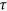. The nuclear spin polarization in *x* and *y* direction can be measured similarly by applying an additional pulse to rotate the nuclear spins before performing the NV measurements^31^.

We demonstrate the basic working principle of this protocol at the hand of the simple example of two Hydrogen atoms at a distance of 1 *Å*, which represents a lower limit to the distance between hydrogen in proteins. In our numerical simulation, we assume an applied magnetic field of 1000 G which allows us to comfortably resolve dipole-dipole energy splitting of about 100 kHz for our choice of physical simulation times for a single measurement of a few milliseconds. The NV center is assumed to be located at a distance of 2 nm to both Hydrogen nuclei. To support selective addressing of the nuclei, the applied magnetic field includes a gradient of 60 G/nm along the axis joining the Hydrogen atoms. This allows for a dominant polarization of a distinct nucleus in the sample, dominant coupling of the NV center to a specific nucleus during the read out and a maximum fidelity of applied pulses for selected nuclei. The applied *π*/2-pulses are implemented by radio-frequency fields with a Rabi frequency of 5 kHz.

**Figure 2:**
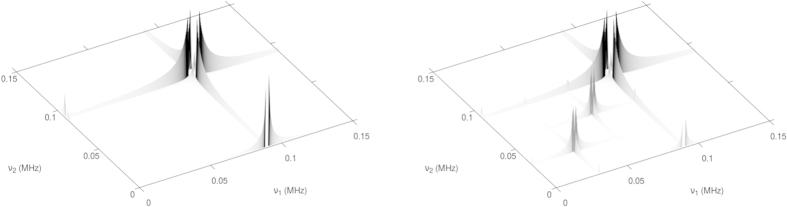
Two-dimensional correlation spectroscopy (COSY) simulation of two Hydrogen atoms arranged at a distance of 1*Å*. As described in the main text, this highly symmetric sample is disturbed by an additional Nitrogen atom, which couples only to one of the two nuclei. **Left:** no application of selection mechanisms during the protocol. Polarization signal is averaged out over the sample. **Right:** The application of selective polarization and control reveals NMR information from single nuclei beyond sample averaged quantities. Features such as the new peaks around 0.03 MHz and 0.06 MHz have also been verified by further simulations that measure the nuclear polarization by direct projection rather than invoking an NV center as sensor.

Figure 2 compares the result of applying the above COSY protocol without (l.h.s.) and with (r.h.s.) the selection mechanisms. The latter case exhibits additional peaks which emerge due to the selective polarization of one of the Hydrogen nuclei. It should be noted that the protocol includes the internuclear coupling (of order 100 kHz) which exceeds the energy shift due to the applied magnetic field gradient (of order 25 kHz) but is nevertheless sufficient to result in clearly identifiable additional peaks in the spectrum. This confirms nicely our expectations, because dropping the selection mechanisms, one may only gain average information for the whole sample. Further simulations suggest, that selective coupling can also lead to effects, where spectra become less crowded by suppressing the unwanted influence of certain nuclei during the read out as will be discussed in more detail for a larger molecule, alanine. In the setting presented so far, we do not identify yet the orientation of the Hydrogen molecule relative to the NV-coordinate system. This can be achieved by exploiting the dependence of the energy splitting due to the internuclear interaction on the relative orientation w.r.t. the externally applied magnetic field as given by


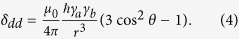


Here *r* is the distance between two nuclei, 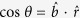 with 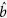 and 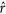 the unit direction vector of the magnetic field and the vector that connects two nuclei respectively and 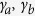 are the nuclear gamma factors. Varying the magnetic field orientation (with three possible directions), one can extract the relative orientation of pairs of nuclei in the sample by fitting Eq. (4) to the splitting of matching pairs. To obtain the values of *r* and *θ*, two independent magnetic field directions are sufficient. From the distances and orientations of the various nuclei relative to the selectively addressed nucleus in the sample, we can learn the spatial structure of its local environment within the molecule.

*Single molecule NMR of Alanine —* Before moving on, we consider the NV-based COSY spectrum of an Alanine molecule in the strong coupling regime as described in the above section. Alanine is one of the smallest amino acids with chemical composition HOOCCH(NH_2_)CH_3_, which carries nuclear spins on the 7 Hydrogen atoms and the single Nitrogen atom. The molecule has a total size of about 0.45 nm and is illustrated in the upper part of Fig. 1. The nuclear coordinates and the internuclear coupling rates can be found in the Supplemental Information.

We assume a shallowly implanted NV center located at a distance of 2 nm to the surface^24^ to measure the polarization of the Hydrogen and Nitrogen atoms by matching the resonance condition for the two species respectively. In this manner we can obtain the corresponding magnetic resonance spectra. Apart from the fact that in the strong coupling regime the NMR signal is enhanced for single molecules the combination with magnetic field gradients opens the possibility to isolate specific parts of the NMR spectrum. Indeed, to further distinguish between specific Hydrogen atoms, we suggest to apply a combination of a gradient field and an external radio-frequency drive to support selective polarization and coupling to individual single Hydrogen atoms by suitable tuning of the NV center to the corresponding transition frequency. This will be particularly effective if the energy mismatch between the Larmor frequency of the NV center and the frequency of the not-to-be-addressed Hydrogen atoms exceeds their coupling strength. For neighboring Hydrogen nuclei in Alanine their distance is of order 1.8 *Å* which results in an interaction of order of around 20 kHz. While it is difficult to exceed this by means of magnetic field gradients our simulations show that the combination of a rf-field with Rabi frequency of 100 kHz with a realistic gradients of 60 G/nm^33^ (resulting in energy shifts in the range [0,45 kHz] depending on the relative orientation of the magnetic field gradient to the positions of the relevant nuclei) we are able to observe significant selective polarisation and coupling.

**Figure 3:**
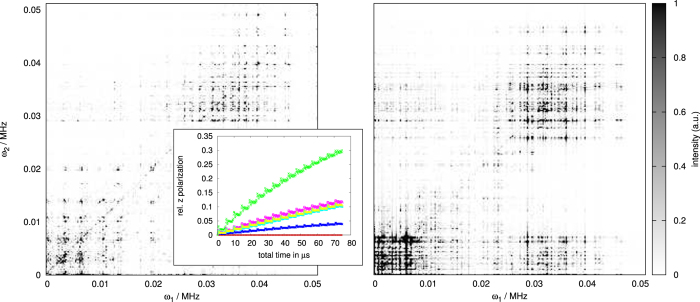
2D COSY NMR spectrum of alanine. **left:** including selective polarization as described in the main text. The splitting occurs due to the hyperfine interaction of the selected nuclear spin with its neighborhood. The peak coordinates are given with respect to the Hydrogen Larmor frequency. **inset:** individual polarization gain for six of 8 nuclear spins in alanine during the initial preparation sequence. The frequency and gradient parameters have been optimized to favour addressing of a specific Hydrogen nuclear spin. Due to the strong interaction between the nuclear spins, nuclear polarization transfer can not be completely suppressed. However, a two to three-fold increase in polarization of the selected nuclear spin as compared to the non-selected ones will result in a remarkable gain in contrast. The same selective coupling efficiency can be expected to hold also during other parts of the protocols that are based on the selective coupling mechanism. **right** : The signal resulting from a fully polarized molecule due to a lack of selective addressing. The spectrum is considerably more congested, clearly highlighting the advantage of dominant addressing of selected nuclear spins.

In order to achieve dominant coupling to a selected hydrogen atom we perform our selective polarization protocol and apply the COSY procedure with the NV center tuned on resonance to the hydrogen transition frequency. This yields the l.h.s of Fig. 3, where the hyperfine coupling can be clearly identified. The decongestion of the spectrum thanks to the dominant addressing of selected nuclei can be observed very clearly by a comparison to a spectrum that one obtains without selective addressing from a fully polarized molecule (see r.h.s of fig. 3). The ability to selectively address a single Hydrogen spin enables the implementation of the NMR protocol for selected atoms in the target molecule and therefore allows for the reconstruction of the molecular environment of the chosen atoms by reading out the hyperfine splitting of each atom individually.

The results of this simulation will be used also for the demonstration of the application of matrix completion in the last section.

*Exploiting entanglement between the NV electron spin and the target nuclei —* The protocols so far exploit the ability to achieve hyperpolarization of the molecule or of some of its constituents. Importantly, however, operating in the strong coupling regime, it is not strictly necessary to polarize nuclear spins, while still retaining a full signal contrast due to the quantum nature of the interaction between the NV center spin and the nuclei. Moreover, we can make use of the strong coupling for the coherent control of both nuclear spins (by means of radio-frequency fields or via the interaction with the NV center) and of the NV center spin (microwave frequency fields) thus allowing for more complex pulse sequences for the simultaneous control of sensor and target.

**Figure 4:**
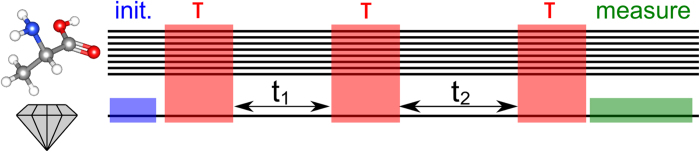
2D NMR spectroscopy pulse sequence with an NV sensor in the strong coupling regime. The nuclei are initially unpolarized, only the NV center is initialized in a *m* = ± 1 state. The final measurement is a projection of the NV center onto its initial state. During the free evolution times *t*_1_,_2_, the NV-nuclear interaction is effectively eliminated, while it is switched on during the periods 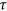.

As an illustrative example, we construct a novel NV-based 2D-spectroscopy scheme by exploiting the strong interaction of nuclei in a target molecule with an NV center spin, see Fig. 4(a). Here we selectively trigger the NV-nuclear spin interaction to generate entanglement between the NV center and the nuclear spins interspersed with free evolution times *t*_1_ and *t*_2_ which in turn allow for an accumulation of the effect of the nuclear Hamiltonian that can subsequently be measured via the NV center. While this scheme appears similar to the well-known COSY sequence it differs from it due to the entanglement that is created between NV center and nuclear spin which in turn allows us to generate the same spectral information as in the case of COSY with fully polarized nuclear spins but now without the need for nuclear spin polarization. Although the actual detection protocol is quite different, this scheme shares sufficient parallels to the COSY scheme from Fig. 1 to transfer the interpretation of peak positions from the original COSY scheme.

For definiteness, we describe the pulse sequence in detail together with the relevant Hamiltonians (see also Fig. 4):

- *Initialization*: the NV center is initialized in a polarized state, while the nuclear spins remain unpolarized (blue bar).
- *NV-nuclear interaction*: the NV center interacts with the nuclei for a time 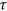 (red bar). In this step the NV center becomes entangled with the nuclear spins. The dynamics is governed by the Hamiltonian eq. (1), where the additional application of an external RF fields effectively decouples the nuclear spins as explained above and again leads to the resonance condition given in equation (2).
- *Free evolution period 1*: the interaction of the NV center with the nuclei is switched off by transfer of the NV center to the *m* = 0 state and if necessary the quantum information may be transferred further to the nuclear spin degree of freedom of the NV center. The nuclear spins precess freely for a time *t*_1_ during which the dynamics is governed by the Hamiltonian

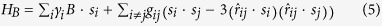

- *NV-nuclei interaction*: The NV center again interacts with the nuclei with the same Hamiltonian eq. (1). The additional entanglement depends on the system state after the previous free evolution time *t*_1_.
- *Free evolution period 2*: the interaction with the NV center is switched off and the nuclear spins precesses freely for a time *t*_2_ under the same Hamiltonian as in the free evolution period 1.
- *NV-nuclear interaction*: A third interaction employing the same dynamics as in the previous two NV-nuclear interaction periods prepares the final measurement.
- *Measurement*: Finally, we perform a projective measurement on the initial state of the NV center spin (green bar).

**Figure 5:**
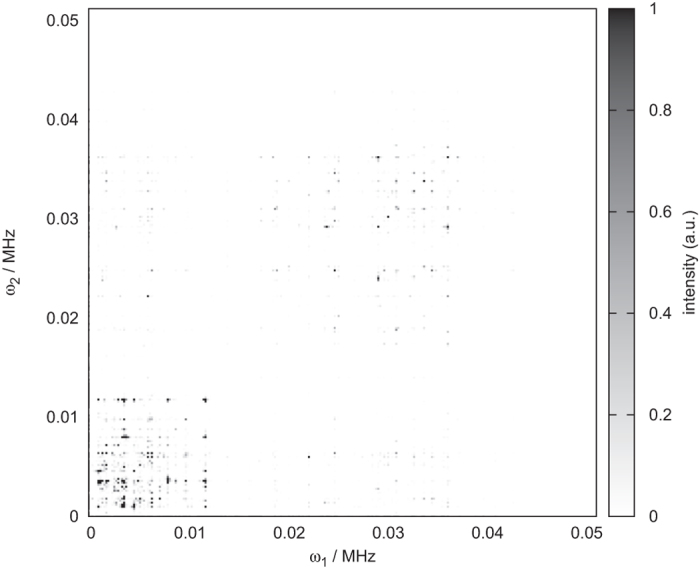
Entanglement based 2D NMR spectroscopy pulse sequence with an NV sensor in the strong coupling regime. Resulting spectrum for an NV center tuned on resonance to a selected Hydrogen transition at a magnetic field of 100 G aligned to the NV axis. *t*_1_,_2_ was varied up to 5 ms, while the time 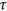 has been chosen around 1.25 ms. Due to the long simulation times, the hyperfine coupling is resolved. The selection mechanism was tuned to another nucleus in this simulation than the COSY equivalent in Fig. 3, which demonstrates the distinguishability.

As illustration of the above 2D NMR protocol with a strongly coupled NV center at a depth of 2 nm we consider Alanine in a magnetic field of 100 G. To preferably address selected nuclear spins we apply an additional gradient field of 60 G/nm, to support dominant entanglement between the NV and selected nuclear spins during the NV interaction periods. The simulated magnetic resonance spectrum is plotted in Fig. 5 from which one can clearly identify thehyperfine splittings. The spectrum is decongested thanks to the dominant coupling to a specific Hydrogen atom supported by strong external magnetic field gradients. This selective coupling is particularly useful for measuring hyperfine splittings of individual atoms, as the coupling strength of the NV center to each Hydrogen atom is nearly identical in the absence of a gradient field and makes more difficult the distinction of the various peaks in the spectra. Therefore, selective coupling to each single atom in the molecule allows for the reconstruction of the molecular neighborhood of atoms and supports the determination of the geometric structure of the molecule. Without individual atomic addressing, the geometric information has to be extracted from just a single spectrum, while the ability of selectively addressing *n* atoms allows for the generation of *n* independent spectra to decongest the spectra.

Assuming that the nuclei are initially not polarized, we have also verified that they will remain nearly unpolarized across the entire pulse sequence, that is we observe NMR signals without relying on any nuclear polarization, which represents a distinct feature as compared with conventional NMR techniques. As above, the application of gradient fields allows for individual coupling even for identical atoms.

## Matrix Completion for an NV-based 2D NMR

For larger molecules, the application of NV-based 2D-NMR suffers from a rapidly increasing experiment effort as a function of the number of nuclei in the target molecule. In this section, we explain that the experimental overhead of NV-based 2D spectroscopy can be reduced significantly by exploiting the technique of matrix completion^25^,^26^ (see for example^27^ for the related but distinct compressive sensing) which exploits two specific aspects of NV-based 2D spectroscopy. First, 2D-spectra generally possess structure which expresses itself in sparseness in a certain basis and, secondly, while the relevant information is represented in Fourier space, the experimental data are taken in time. We will begin by clarifying why these two aspects are important and how they are going to be being used.

*Background —* For a given matrix *A* we denote with *σ*_*i*_ its descendingly ordered singular values, that is the diagonal entries of the matrix D in 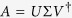 where *U* and *V* are unitaries. If these singular values are close to zero for indices *i* > *r*, one obtains a high fidelity approximation 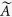 with rank *r* for the matrix *A* by


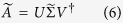


where 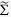 is a low rank version of 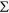, as defined by


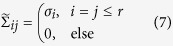


The idea of matrix completion is to reconstruct the matrix *A* by finding a low rank approximation 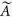 based on the knowledge of a few entries from a random sample set {(*i*, *j*)} = Ω such that 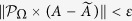, where 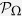 is a boolean matrix that indicates whether (*i*, *j*) is in Ω or not and 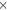 means elementwise multiplication.

It is important to note that generally the basis in which one takes the random samples will affect the reconstruction efficiency. Indeed, sampling a matrix with just one nonzero entry, i.e. a very sparse matrix, will tend to yield only zeros upon random sampling of the matrix entries and hence any reconstruction algorithm must conclude that all the entries of the matrix are in fact zero. This situation differs considerably when we take the discrete Fourier transform of this matrix and sample the result. Now sampling even a small number of entries will yield useful information about the structure of the matrix and indeed it becomes possible then to reconstruct this Fourier transformed matrix (and hence also the original) from a small number of sampled entries (for a *n* × *n* matrix the number of required samples scales as *rn* ln *n* where 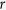 is the singular value rank of the data matrix). Indeed, for a matrix that is sparse in some basis {|e_*i*_〉} (i.e. most of its entries vanish or are negligible) it is in this sense optimal to sample the matrix in the Fourier transformed basis {*F*|e_*i*_〉} (see e.g^34^. for a rigorous treatment that provide the mathematical foundation for these observations).

It is essential that the data matrices that one obtains from NV-based 2D-NMR tend to be approximately sparse as their entries are concentrated on the diagonal and around the off-diagonal positions that indicate coupling between those diagonal elements. From these arguments and observations it becomes transparent that matrix completion is ideally suited to support NV-based NMR spectroscopy as the desired information is represented in frequency while the data are taken in time, that is we sample the desired information in a basis that is Fourier transformed with respect to the information basis. We will make use of this fact in the remainder of this section.

*Application —* We simulate a subset of the entries *S*_*t*_ corresponding to randomly chosen *t* = (*t*_1_,*t*_2_) in the time-domain signal, which are those data that would be measured in real experiments. The matrix completion algorithm, whose run time is negligible compared to the savings in measurement time^35^, will reconstruct the 1024 × 1024 time domain matrix *S*_*t*_ out of the subsampled entries. We will see that in the frequency domain, the hyperfine structure can then be identified from the reconstructed matrix.

**Figure 6:**
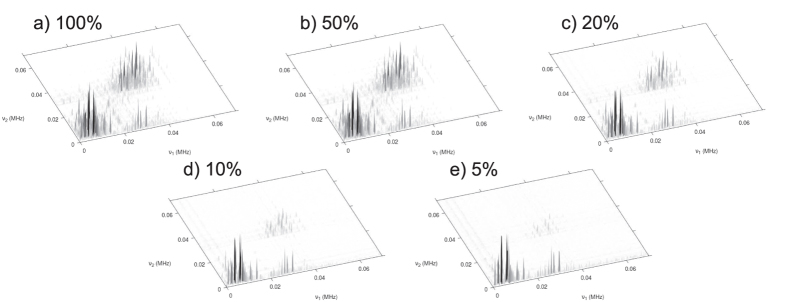
The magnetic resonance spectra of a simulated Alanine molecule based on a NV-based COSY NMR and matrix completion. Comparison of the distribution of the peaks from the spectrum shown in Fig. 3 after matrix completion for different sample rates. (***a***) full data. (***b***) 50% data sample. (***c***) 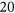
% data sample. (***d***) 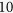
% data sample and (***e***) 5% data sample. One can clearly see, how weak contributions diminish, while strong contributions remain nearly untouched even at very low sampling rates.

As a comparison, we apply the completion algorithm to the Alanine COSY spectra generated above and plot in Fig. 6(a) the spectrum obtained from the complete set of measurements, namely based on a 100% data sample. In contrast, Fig. 6(b) shows the same as Fig. 6(a), for different sample rates. It can be seen that we are able to identify the relevant splitting structure in the Hydrogen signal even with about 20% data sample. Hence we expect a remarkable time saving due to sub-sampling of both experimental and simulated data for sufficiently large matrices.

## Conclusions and Discussions

The shallow implantation of NV centers in diamond allow for a new magnetometry regime in which the coupling of the NV center to nuclei is comparable or larger than the inter-nuclear coupling, in rapid departure from the standard regime in which nuclear magnetic resonance in bulk is situated. We have demonstrated that this strong coupling regime offers new opportunities for single molecule nuclear magnetic resonance spectroscopy that can reduce the experimental requirements both by relaxing the need for nuclear spin polarization while retaining a significant signal, by decongesting the NMR spectra thanks to individual addressing for polarization and readout of nuclear spins and by reducing significantly the number of measurements required to recover the 2D spectrum thanks to the application of matrix completion, a method from signal processing. In numerical studies we have applied these ideas to a specific example of a small amino acid, alanine to demonstrate the feasibility and potential of this approach. We expect that the combination of these approaches may be applied beyond this example to extend to the study of membrane proteins whose structure and dynamics are of considerable importance in biology and medicine.

## Additional Information

**How to cite this article**: Kost, M. *et al.* Resolving single molecule structures with Nitrogen-vacancy centers in diamond. *Sci. Rep.* **5**, 11007; doi: 10.1038/srep11007 (2015).

## Supplementary Material

Supplementary Information
