# Supplementary material for: Resolving single molecule structures with Nitrogen-vacancy centers in diamond
Source: Sci Rep. 2015 Jun 5;5:11007. doi: 10.1038/srep11007 (PMC4457023; doi:10.1038/srep11007)
Supplement: Supplementary Information [file srep11007-s1.pdf]

Supplementary Information

The simulation of the 2D NMR spectra of Alanine is based on the Hamiltonians eqs. (1-3) of the main text. Of particular importance are the values that determine the internuclear interactions as well as the interaction strengths between NV center and nuclei. These parameters required to infer these quantities are presented in this section.

The positions of the nuclei in the Alanine sample used in the simulations are given in table I.

Table I: positions of the nuclei in the alanine simulations.

index	species	r_x/nm	r_y/nm	r_z/nm
0	NV	0.50000	1.50000	1.20000
1	N	0.162815	-0.01544847	-0.04377297
2	H	-0.1443138	0.1366065	-0.04377297
3	H	0.007342323	0.1854949	-0.1357687
4	H	0.007342323	0.1854949	0.04822277
5	H	-0.0170428	-0.06919056	-0.1333172
6	H	0.1882076	-0.1132707	-0.04377297
7	H	0.1929836	0.02212539	-0.1326101
8	H	-0.1538646	-0.12967	0.2265698

The relative distances between the relevant nuclei are given in table II. It should be noted that the typical distance between hydrogen nuclei is lower bounded by $1.8\mathring{A}$ leading to typical interactions strength not exceeding 20kHz.

Table II: Relative distance between the nuclei in nm. The indices refer to the labelling in table I.

	0	1	2	3	4	5	6	7	8
0		1.98	1.95	1.93	1.81	2.12	2.06	2.01	2.00
1	1.98		0.34	0.27	0.27	0.20	0.10	0.10	0.43
2	1.95	0.34		0.18	0.18	0.25	0.41	0.36	0.37
3	1.93	0.27	0.18		0.18	0.25	0.36	0.24	0.50
4	1.81	0.27	0.18	0.18		0.31	0.36	0.30	0.39
5	2.12	0.20	0.25	0.25	0.31		0.22	0.22	0.38
6	2.06	0.10	0.41	0.36	0.36	0.22		0.16	0.43
7	2.01	0.10	0.36	0.24	0.30	0.22	0.16		0.52
8	2.00	0.43	0.37	0.50	0.39	0.38	0.43	0.52	

The dipole-dipole-interaction between nucleus i and nucleus j is given by

$$H_{ij} = g_{ij} \left(3 \left(\mathbf{r}_{ij} \mathbf{s}_i \right) \left(\mathbf{r}_{ij} \mathbf{s}_j \right) - \mathbf{s}_i \mathbf{s}_j \right), \tag{1}$$

where $\mathbf{s}_i$ is the spin operator and $\mathbf{r}_{ij}$ is the distance vector between the nuclei. The spatial structure of the Alanine molecule leads to the coupling constants

$$g_{ij} = \frac{\mu_0 \hbar^2 \gamma_i \gamma_j}{4\pi r_{ij}^3} \tag{2}$$

with μ_0 being the vacuum permeability and γ_i being the gyromagnetic ratio of the corresponding nucleus. Numerical values for the coupling constants are given in table III.

Table III: Coupling constants for the dipole-dipole-interaction between the nuclei in kHz. The indices refer to the labelling in table I.

	0	1	2	3	4	5	6	7	8
0		1.01	10.58	10.86	13.19	8.25	9.02	9.68	9.76
1	1.01		0.30	0.61	0.61	1.35	11.78	11.78	0.15
2	10.58	0.30		19.27	19.27	6.98	1.66	2.42	2.19
3	10.86	0.61	19.27		19.27	7.16	2.54	7.93	0.92
4	13.19	0.61	19.27	19.27		3.88	2.54	4.17	1.92
5	8.25	1.35	6.98	7.16	3.88		10.09	9.99	2.02
6	9.02	11.78	1.66	2.54	2.54	10.09		28.23	1.44
7	9.68	11.78	2.42	7.93	4.17	9.99	28.23		0.84
8	9.76	0.15	2.19	0.92	1.92	2.02	1.44	0.84	
